# Role of esculin antioxidant on remediation of lead toxicity on flax growth and yield components

**DOI:** 10.1186/s12870-025-06870-7

**Published:** 2025-07-03

**Authors:** Heba M. Hassan, Nessrin Sh. Bakhoum, Mona G. Dawood, Mervat S. Sadak, Hemmat I. Khattab

**Affiliations:** 1 Botany Department, Faculty of Science, Ain Shams University, Cairo, 11566 Egypt; 2 Nuclear Materials Authority, Cairo, Egypt; 3 Botany Department, Agricultural and Biological Research Institute, National Research Centre, 33 El-Behouth Street, Dokki, P. O. 12622, Giza, Egypt

**Keywords:** Esculin, Lead toxicity, Flax, Defense mechanism, Yield quality

## Abstract

**Background:**

Esculin, a natural coumarin glucoside with antioxidant properties, is a secondary metabolite found in various plant species and has been traditionally valued for its medicinal benefits. This study focused on evaluating the ability of esculin to enhance flax plant tolerance to lead (Pb) toxicity.

**Methodology:**

Flax plants were irrigated with either distilled water or a lead solution (200 mg/L) and subsequently treated with foliar sprays of esculin at concentrations of 0, 50, and 100 mg/L.

**Results:**

The findings revealed that esculin application significantly improved the plants’ resilience to Pb toxicity. It reduced the levels of reactive oxygen species (ROS), malondialdehyde (MDA), and electrolyte leakage (EL), while improving the membrane stability index (MSI). Esculin effectively alleviated oxidative stress by boosting the activity of antioxidant enzymes such as superoxide dismutase (SOD), catalase (CAT), peroxidase (POX), phenylalanine ammonia-lyase (PAL), and tyrosine ammonia-lyase (TAL), in addition to increasing total phenols and proline content. Furthermore, esculin contributes to maintaining membrane integrity by reducing sodium (Na) uptake and increasing the absorption of potassium (K), magnesium (Mg), phosphorus (P), and calcium (Ca). It also minimizes Pb accumulation in the plants, further mitigating lead toxicity. These effects were associated with improved photosynthetic pigment concentrations, higher levels of soluble sugars (TSS), and an increase in free amino acids (FAA) under lead stress conditions. The application of esculin enhanced key agronomic traits, including shoot and root growth, fresh and dry biomass, seed yield per plant, and 1000-seed weight. Additionally, seed quality improved, with increased oil, carbohydrate, and protein content under Pb stress conditions.

**Conclusion:**

In summary, esculin glycosides is a novel approach that effectively reduces lead toxicity and enhances stress tolerance in flax, offering a promising strategy for managing heavy metal stress in crops.

## Introduction

Chemical pollution, particularly from heavy metals, is a major factor contributing to the degradation of the biosphere [1]. Heavy metals, typically defined as elements with a density greater than 5 g/cm³, include 53 of the 90 naturally occurring elements [2]. Among these, lead (Pb) is an amphoteric trace element and is recognized as the second most toxic heavy metal [3].

Despite its presence in air, water, and soil, Pb contamination in soil poses the greatest threat to human health and ecosystems, as extensively studied [4, 5]. Meanwhile, plants respond differently to Pb contamination. Hyperaccumulator plants tolerate and accumulate high Pb levels without visible damage [6], while accumulator plants display toxicity symptoms when exposed to high concentrations [7]. Sensitive plants fail to grow in contaminated environments [8].

Lead adversely affects plant growth and development by reducing germination rates, seedling growth, root and shoot length, and biomass [9–12]. High Pb levels disrupt metabolic processes, causing mineral imbalances by interfering with the uptake of essential ions (e.g., Fe³⁺, Mn, Ca, Mg, Zn, K⁺, Cu, and NO₃⁻) [13]. At the cellular level, Pb inhibits enzyme activity by interacting with SH and COOH groups essential for enzyme function and stability, ultimately disrupting metabolic pathways and potentially causing cell death [14, 15].

Lead (Pb) toxicity is a significant environmental stressor that impairs plant growth by inducing oxidative stress. This stress arises from the excessive production of reactive oxygen species (ROS), including hydroxyl radicals (·OH), hydrogen peroxide (H_₂_O_₂_), singlet oxygen (¹O_₂_), and superoxide anions (O₂⁻) [16, 17]. As a result, excessive ROS levels cause oxidative damage to cellular components, notably increasing lipid peroxidation a key marker of oxidative stress. For instance, Verma and Dubey [17] reported significantly elevated lipid peroxidation in *Oryza sativa* seedlings exposed to 500 and 1000 µM Pb(NO₃)₂ in sand culture.

Berry [18] classified plant responses to heavy metals into three categories: avoidance, detoxification, and biochemical tolerance, which involve exclusion mechanisms and the activation of robust physiological defense systems. To counteract metal oxidative stress, plants rely on both enzymatic antioxidants such as catalase, peroxidase, superoxide dismutase (SOD), ascorbate peroxidase (APX), and glutathione reductase (GR) and non-enzymatic antioxidants, including phenolic compounds, glutathione, and ascorbic acid [17].

Esculin (6,7-dihydroxycoumarin 6-O-glucoside) is a naturally occurring compound found in various plant species, including *Taraxacum*, *Cichorium*, horse chestnut (*Aesculus hippocastanum*), and *Fraxinus ornus* [19–22]. Known for its strong antioxidant and anti-inflammatory properties, esculin is also a major bioactive component of the traditional Chinese medicine Qinpi, derived from *Fraxinus* bark [23, 24]. Esculin enhances the plant’s antioxidant defense by stimulating the activity of key enzymes such as superoxide dismutase (SOD), glutathione reductase (GR), and glutathione peroxidase (GPx), thereby reducing the accumulation of reactive oxygen species (ROS) and malondialdehyde (MDA) [25]. Recent findings suggest esculin’s potential application in agriculture, as it has been shown to improve salinity stress tolerance in flax (*Linum usitatissimum* L.) plants [26].

Flax (*Linum usitatissimum* L.) is a widely cultivated, economically significant crop known for its dual-purpose utility in fiber and oil production [27]. The global demand for flax has been increasing, driven by intensified research efforts and the recognition of its broad industrial and nutritional applications [28]. Flax fiber exhibits superior mechanical properties as being approximately three times stronger than cotton, and is characterized by its straightness, softness, durability, high water absorbency, and swelling capacity, making it highly suitable for textile manufacturing, particularly linen production [29]. Flax seeds are an abundant source of bioactive constituents, including high-quality proteins, lignans, and alpha-linolenic acid (ALA), which contribute to their nutritional and therapeutic value for both human and animal diets [30, 31]. Consequently, flax seeds are extensively utilized in oil extraction processes, as components of functional foods, and in the formulation of animal feed [32, 33]. Despite its agronomic and industrial relevance, improving flax yield and quality under abiotic stress conditions remains a key challenge. Therefore, the present study aims to evaluate the efficacy of esculin, a plant-derived antioxidant compound, in enhancing flax growth and yield performance under lead (Pb) toxicity stress.

## Materials and methods

Flax seeds were obtained from the Agricultural Research Centre, Giza, Egypt.

### Experimental procedure

A pot experiment was conducted during the winter season of 2021/2022 (from November 15, 2021, to May 9, 2022) to evaluate the effects of foliar application of esculin on flax (*Linum usitatissimum* L.) plants subjected to lead (Pb) stress. Lead nitrate [Pb(NO₃)₂] was applied 45 days after sowing at a concentration of 200 mg Pb L⁻¹. Esculin treatments (50 and 100 mg L⁻¹) were applied as foliar sprays on the 30th and 37th days after sowing. Throughout the experimental period, the average daily temperature was 15.4 ± 1.0 °C, with nighttime temperatures averaging 13.1 ± 1.6 °C. Relative humidity ranged from 22.1 to 59.2%, with a mean value of 49.7 ± 9.2%.

Before the experiment, composite soil samples were collected and analyzed for physicochemical properties following standard procedures described by [34, 35]. To improve aeration and drainage, clay soil was mixed with sand at a volumetric ratio of 3:1. The final soil composition was composed of 1.4% coarse sand, 31.7% fine sand, 39.6% silt, and 27.3% clay. The soil had an electrical conductivity (EC) of 1.82 dS m⁻¹, pH 7.5, 1.93% organic matter content, and calcium carbonate (Ca CO₃) content of 7.88%. Available nitrogen, phosphorus, and potassium were 45.6, 7.8, and 415.0 mg kg⁻¹, respectively. The background concentration of lead in the soil was 8.18 mg Pb kg⁻¹.

To maintain consistent Pb levels, lead nitrate was applied in equal volumes at regular intervals, ensuring a constant concentration of 200 mg Pb L⁻¹ throughout the experiment. Fertilizers were applied at rates of 5 g superphosphate, 2.5 g potassium sulfate, and 6 g urea per pot. The experiment was arranged in a completely randomized design (CRD) with three replicates per treatment.

### Experimental design, growth and yield & yield components

The pot experiment was conducted in the greenhouse of Botany Department, National Research Center. Experimental design was complete randomized blocks design. Flax variety Letwania-9 seeds were chosen of the same size and colour and sterilized for nearly 2 min with 1% sodium hypochlorite after gently washed by distilled water. Ten identical air-dried seeds were planted in plastic pots at thirty mm depth 30 mm, in approximately seven kg clay soil. Following the guidelines provided by Egyptian Ministry of Agriculture and Land Reclamation all guidelines practices related to flax production were followed out. After plant emergence, flax seedlings were thinned ten days after sowing (DAS), and five plants per pot were left. The experiment comprised two factors. The first factor included two lead nitrate levels namely L0 and L 200 mg L^−1^. The second factor involved the foliar application of different concentrations of esculin (0, 50, 100 mg L^−1^) at 30 and 45 days. A preliminary germination experiment was conducted using esculin at concentrations 0.0, 25, 50, 100, 150, 200 mg L^−1^. The appropriate concentrations of esculin were selected based on preliminary observations of plant growth characteristics. To prevent esculin contamination of the soil during foliar application, the surface of each pot was carefully covered prior to spraying. A surfactant, Tween-20 (0.1% v/v), was added to the esculin solution to enhance foliar absorption and facilitate efficient penetration into leaf tissues. All foliar treatments were applied early in the morning, before 9:00 a.m., using a hand-held sprayer with adequate pressure to ensure a fine mist and uniform droplet distribution. Plants were sprayed thoroughly from all directions to achieve full leaf coverage. The experimental design included four treatments: (1) control (0 L + 0 E), (2) 200 mg Pb L^−1^, (3) L 200 mg L^−1^ + E 50 mg L^−1^, (4) L 200 mg L^−1^ + E 100 mg L^−1^.

### Measurements

Plant samples were taken after 75 days to assess growth criteria in terms of shoot length (cm), fresh and plant dry weight (g plant^−1^), root length (cm), and root fresh weight (g). Additionally, some chemical analyses were performed. Once the flax plants reached full maturity, they were uprooted and left to dry completely on the ground. The capsules were carefully taken out. At harvest, random samples of ten guarded plants in each plot were measured for plant height (cm), fruiting zone length (cm), technical stem length (cm), number of fruiting branches/plant, number of capsules/plant, biological yield/plant (g), seed yield/plant (g), and 1000 seeds wt (g). Some biochemical analyses of the yielded seeds were done, such as oil percentage, carbohydrates, and protein.

### Calculation of the flax tolerance index (TI)

The tolerance index was defined as the ratio of the plant height, dry weight or fresh weight of the flax plant grown under lead pollution conditions to that grown in the lead free condition. The tolerance index was calculated following the formula given by Shi and Cai [36].

$$\:\text{T}\text{I}=\frac{\text{M}\text{e}\text{a}\text{n}\:\text{s}\text{h}\text{o}\text{o}\text{t}\:\text{l}\text{e}\text{n}\text{g}\text{t}\text{h}\:\text{o}\text{r}\:\text{D}\text{r}\text{y}\:\text{w}\text{e}\text{i}\text{g}\text{h}\text{t}\:\text{o}\text{r}\:\text{F}\text{r}\text{e}\text{s}\text{h}\:\text{w}\text{e}\text{i}\text{g}\text{h}\text{t}\:\text{u}\text{n}\text{d}\text{e}\text{r}\:\text{P}\text{b}\:\text{s}\text{t}\text{r}\text{e}\text{s}\text{s}}{\text{M}\text{e}\text{a}\text{n}\:\text{s}\text{h}\text{o}\text{o}\text{t}\:\text{l}\text{e}\text{n}\text{g}\text{t}\text{h}\:\text{o}\text{r}\:\text{D}\text{r}\text{y}\:\text{w}\text{e}\text{i}\text{g}\text{h}\text{t}\:\text{o}\text{r}\:\text{F}\text{r}\text{e}\text{s}\text{h}\:\text{i}\text{n}\:\:\text{a}\text{b}\text{s}\text{e}\text{n}\text{c}\text{e}\:\text{o}\text{f}\:\text{P}\text{b}\:\text{s}\text{t}\text{r}\text{e}\text{s}\text{s}\text{s}\:}X100$$

### Biochemical analysis

#### Determination of hydrogen peroxide (H_2_O_2_) and superoxide radicles (O_2_^−•^)

The Hydrogen peroxide level was determined calorimetrically according to Yu [37]. H_2_O_2_ was extracted from plant tissues by using acetone. Titanium reagent and ammonium were added to the extract and dissolved in sulphuric acid (1 M). The intensity of the yellow color of the supernatant was measured at 415 nm. Superoxide anion radicals were determined by using nitroblue tetrazolium (NBT) according to the method described by Doke [38]. The reaction medium contains 625 µL phosphate buffer (pH 7.6), 25 µL riboflavin (0.2 mg/mL), 50 µL EDTA (12 mM), 25 µL NBT (1 mg/mL) mixed with 25 µL of sample. The reaction mixture was illuminating for 10 min. The superoxide radical level was assayed spectrophotometrically by the increase in the amount of the absorbance at 580 nm.

#### Determination of lipid peroxidation

The level of lipid peroxidation was measured by determining the malondialdehyde (MDA) content using the method of Hodges [39]. A sample (200 mg) was homogenized in 10 ml of 0.1%trichloroacetic acid (TCA). 1 ml of the supernatant was centrifuged at 15,000×g for 20 min to aliquot of the supernatant 4.0 ml of 0.5% (**w/v)** thiobarbituric acid (TBA) in 20% TCA and 10 µl of butylated hydroxyl toluene (BHT) (4% in ethanol) were added. The mixture was heated at 95 °C for 30 min and then quickly cooled in an ice bath, centrifuged at 10,000×g for 10 min, and then the absorbance of the supernatant was recorded at 532 nm by spectrophotometer (VEB Carl Zeiss). The value for non-specific absorption at 600 nm was subtracted. The TBARS content was calculated using its absorption coefficient of 155 nmol^−1^ cm^−1^ and expressed as nmol (MDA) per g fresh weight.

#### Determination of membrane stability index MSI (µS/m)

The electrolyte leakage was measured according to the method recorded by Karimi [40]. Leaf discs from each subgroup were excised and rinsed thoroughly with distilled water. Samples were then transferred to tubes with 20 ml double distilled water. The electrical conductivity (E0) of the solution was immediately measured using an electrical conductivity meter (DDSJ-308 A, Shanghai) at 25 °C. The tubes were incubated at 30 °C for 30 min, and the electrical conductivity (E1) measured again. Subsequently, the tubes were placed in boiling water bath for 20 min. and the electrical conductivity (E2) read after the tubes had cooled to 25 °C. The electrolyte leakage percentage (EL%) of leaf cells was calculated in accordance with the following equation:

$$\text{EL} \% = (\text{E1}-\text{E0}) / (\text {E2}-\text{E0}) \times 100$$

#### Determination of membrane leakage (ML)

Membrane leakage was determined according to Vahala [41]. Samples from flag leaves (0.2 g each) were cut into uniform size discs, washed three times with distilled water to remove surface adhered electrolytes, and placed in 20 ml distilled water followed by centrifugation for 80 min at 300 rpm. Electric conductivity divided by fresh weight as a percentage represents the total ion leakage.

#### Assay of Lipoxygenase (LOX) activity

Lipoxygenase (EC 1.13.11.12) activity was estimated according to Doderer [42] by measuring the rise in absorbance at 234 nm utilizing linoleic acid as a substrate. The activity was calculated using the extinction coefficient (25 mM^−1^ cm^−1^) and expressed as unit (1 n mol of substrate oxidized per minute) per mg enzyme protein.

#### Determination of Na^+^, P, K^+^, Ca^2+^, Mg^2+^ and Pb content

Samples were digested using an acid mixture consists of 750 ml of concentrated nitric acid (69%), 150 ml of concentrated sulphuric acid (98%) and 300 ml of 60 to 62% perchloric acid. The solubilized trace metals were then filtered and diluted up to a known volume with deionized H_2_O [43]. A blank sample was digested under the same conditions. The trace metals were estimated by using inductively coupled plasma-mass spectrometry (ICP-MS) model 7500 of Agilent, USA. ICP-MS.

#### Determination of photosynthetic pigments

The photosynthetic pigments in terms of chlorophyll *a* (Chl *a*), chlorophyll *b* (Chl *b*), and carotenoids were extracted and estimated based on the method of Metzner et al., [44]. One gram of fresh leaves was grinded in 85% aqueous acetone for 5 min. Then centrifuged, and complete the supernatant up to 100 mL with 85% acetone. The extinction was measured against a blank of pure 85% aqueous acetone at three different wavelengths (452.5, 644, and 663 nm) by using a spectrophotometer.

#### Determination of Indole acetic acid (IAA) content

The content of IAA was estimated depending on the method of Larsen [45]. The fresh plant tissue was ground in cold ethanol (85%) and the extraction was completed using an electric stirrer in 85% ethanol at about 0 °C. Then, the filtrate was concentrated under a vacuum at 20–25 ◦C to a few mills. Finally, IAA was estimated by using gas liquid chromatography (GLC) in GVC pye Unicam gas-liquid chromatograph equipped with a dual flame ionization detector and dual channel recorder.

#### Assay of enzymes activities

Enzyme extractions were collected following the method described by Mukheriee and Choudhuri [46]. Fresh tissue was immersed in liquid nitrogen, then grinded by pestle in a mortar. Then, the powder was added to 10 ml of 100 mM phosphate buffer (KH_2_PO_4_/K_2_HPO_4_, pH 6.8), centrifuged at 20,000 x g for 20 min. The supernatant was completed up to known volume with the same buffer and used as an enzyme extract.

#### Assay of superoxide dismutase activity

Superoxide dismutase (SOD) (EC 1.12.1.1) activity was assayed at 560 nm by nitro-blue-tetrazolium (NBT) reduction method using spectrophotometer [47]. The reaction mixture (3 ml) was composed of 150 µ riboflavin (13 µM), 250 µ NBT (63 µM), 2.5 ml methionine (13 µM), 50 µ phosphate buffer (50 mM, pH 7.8), and 50 µ enzyme extract. One unit of SOD activity (1 nmol of substrate oxidized per minute) was described as the quantity of enzyme protein required for prevention of the 50% decrease of NBT.

#### Assay of catalase activity

Catalase (CAT) (EC 1.11.1.6) activity was determined spectrophotometrically by following the decrease in absorbance at 240 nm [47]. The mixture (3 ml) contained 1.9 ml phosphate buffer (50 mM, pH 7.0), 100 µl enzyme extract, and 1 ml0.3% H_2_O_2_. The reaction was initiated by adding enzyme extract. One unit of CAT activity was defined as the 0.01deduction in absorbance at 240 nm per minute.

#### Assay of peroxidase activity

Peroxidase (POX) (EC 1.11.1.7) activity was determined by Kumar and Khan [48]. 0.5 ml of the enzyme extract, 2 ml of 0.1 M phosphate buffer (pH 6.8), 1 ml of 0.01 M pyrogallol and 1 ml of 0.005M H_2_O_2_ were mixed. The solution was incubated for 5 min at 25 °C after which the reaction was terminated by adding 1 ml of H_2_SO_4_ (2.5 N). Purpurogallin amount formed was determined by measuring the absorbance at 420 nm using spectrophotometer. The enzyme activity was calculated as mM of H_2_O_2_/g FW/min.

#### Determination of PAL and TAL activity

The plant tissue (0.5 g) was homogenized in 3 mL ice-cold sodium borate buffer (pH = 8.8) containing 1.4 mM 2-mercaptoethanol and 0.1 g of polyvinyl pyrrolidone. Then the mixture was centrifuged at 10,000 rpm for 25 min. PAL activity was determined by mixing an aliquot (0.1 mL) with 0.1 M tri-sodium borate buffer (pH 8.5) and 0.5 mL of 12 mM L-phenylalanine. The final volume was completed to 3 mL by deionized water and incubated at 30 ◦C for 30 min. Then the increase in absorbance was recorded at 270 nm for 5 min. TAL activity was determined by measuring the amount of p-coumaric acid at 333 nm [49].

#### Determination of total phenolic compounds

The method used in total phenols estimation was that of Malik and Singh [50],. The extraction was carried out by 80% methanol, then was evaporated, the residue was dissolved in distilled water. The phenol extract (0.5 mL) was mixed with 0.5 mL of Folin–Ciocalteu reagent (for phenol). The mixture was kept to stand for 3 min, then one mL of sodium carbonate (25%) was added and it was kept to stand for 60 min at room temperature. Finally, the absorbance was determined at 725 nm by spectrophotometer (Spectronic 601, Milton Roy Company, Ivyland, PA, USA).

#### Determination of total carbohydrates

Total soluble sugars (TSS) were extracted and determined by the method described by Chow and Landhausser [51]. A known weight (1 g) of dry tissue was homogenized in 10 ml of 80% ethanol (v/v) at 25 °C with periodic shaking (overnight), and centrifuged at 6000×g. The supernatant was dried then dissolved in a known volume of distilled water. TSS were analyzed by mixing 0.1 ml of the extract with 3.0 ml freshly prepared anthrone (150 mg anthrone + 100 ml 72% H_2_SO_4_) and boiled in water bath for 10 min, cooled and then the absorbance was recorded at 625 nm by using Spectro-cololourimeter using glucose standard for calibration and expressed as mg glucose/g dry weight.

#### Determination of total free amino acids

The total free amino acid content was determined following the procedure of Sorrequieta et al., [52]. A 0.1 mL aliquot of the extract was combined with 1.6 mL of an ethanol/acetone mixture (1:1, v/v), 0.1 mL of phosphate buffer (0.5 M, pH 6.5), and 2 mL of ninhydrin reagent. The mixture was incubated in a boiling water bath for 20 min, followed by cooling to room temperature. The volume was then adjusted to 10 mL with methanol. The optical density was measured at 580 nm using a spectrophotometer (Spectronic 601, Milton Roy Company, Ivyland, PA, USA).

#### Determination of free proline

Proline content was determined by Kalsoom [53]. 0.5 g of dry leaves was ground in 10 ml of 3% sulphosalicylic acid and centrifuged at 10.000×g for 10 min. The supernatant (2 ml) was mixed with 2 ml of freshly prepared acid ninhydrin reagent. The mixture was incubated at 90ºC in a water bath for 30 min. Reaction was terminated in cooled in the ice bath. The reaction was extracted by adding 5 ml of toluene and vortex and the process was done for 15 s and lasted for 20 min in the dark to separate the toluene and aqueous phases. The toluene phase was collected, and the absorbance of the obtained color was read at 520 nm using proline as standard and expressed as µg g^−1^ fresh weight.

### Biochemical analysis of seed

#### Determination of oil

The oil of seeds was extracted by using Soxhlet apparatus as described by Das [54]. The dried grinded powdered seeds (4 g) were shaken with iso-propanol: chloroform (1:1) overnight. The solvent was evaporated in the presence of reduced pressure of CO_2_ in a rotary evaporator at 40 ^o^ C until the solvent was removed. The residue of lipids is dissolved in chloroform: methanol (2:1 v/v) and the total pure oil was weighed after evaporation of chloroform.

#### Determination of total carbohydrates

Total carbohydrates were estimated according to Albalasmeh [55]. A known weight of dried tissue (0.2–0.5 g) was mixed with 10 mL of sulphuric acid (1 N) and kept overnight in an oven at 100ºC. The solution was then filtered and completed to a known volume. Then one mL of sugar solution was mixed with 1mL of 5% aqueous phenol solution followed by 5.0 mL of concentrated sulphuric acid. Then the tubes were thoroughly shaken for 10 min and kept in a water bath at 23–30ºC for 20 min. The absorbance was recorded at 490 nm.

#### Determination of total protein

The total protein concentration was determined according to the method described by Bradford [56] with bovine serum albumin as a standard. A known weight (2 g) of the sample was ground with 5mL of phosphate buffer (pH 7.6). Then 30µL of the sample was mixed with 70 µL of distilled followed by 2.9 mL of Coomassie Brilliant Blue solution. The samples were kept at room temperature for 5 min and the absorbance was measured at 600 nm.

### Fiber analysis

At full maturity, flax-defoliated plants were collected for the retting process as described El - Hariri [57]. Then the separated fibers were combed with a special comb as reported by El - Hariri (1968). The fiber quality as evident in terms of fiber length (cm) fiber weight/plant (g), straw yield/plat (g) and fiber% was determined in the obtained fibers.

### Cellulose determination

Cellulose% was determined according to the method of Updegraff [58] where the fiber dissolved in acetic acid and nitric acid to remove lignin, hemicellulose, and xylans. The cellulose was reacted with anthrone in sulfuric acid. The obtained colored compound is assayed spectrophotometrically at 635 nm.

### Lignin determination

The fiber lignin% was determined by the method described by Collings [59] Fiber was dispersed in 0.5% ammonium oxalate, boiled for 2 h, and filtered. The extracted fiber was re-suspended in 1% acetic acid at 70 °C, sodium chlorite (1.25 g) was added and lignin was oxidized for a maximum of 45 min. Oxidation was stopped by adding ascorbic acid, and the suspension was filtered and dried at 60 °C for 4 h. The difference in weight was lignin.

### Statistical analysis

Analyses of variance (ANOVA) for all data presented in this investigation were calculated using SPSS v20.0 (SPSS Inc., Chicago, USA) analyzing software. Statistical significances of the means were compared with Duncan’s test at *p* ≤ 0.05 levels [60], the standard error (SE) of the means presented in tables, and the figures are means ± SE (number of replicates = 3).

## Results

### Reactive oxygen species, MDA, MSI%, EL% and LOX activity

The results of this study showed a significant increase in ROS content such as H_2_O_2_ and O_2_^−•^ in Pb-stressed flax plants as compared with unstressed control (Table 1). Also, Pb -stress significantly increased MDA content, % EL as well as LOX activity (Table 1). In the contrary, % MSI significantly reduced under Pb stress. Treatment with esculin significantly reduced the accumulation of H_2_O_2_, O_2_^−•^, MDA contents and LOX activity in stressed flax plants. The highest decrease percentage was observed at higher concentrations of esculin 21.3%, 21.6%, 24.7% and 22.3% for H_2_O_2_, O_2_^−•^, MDA contents and LOX activity from control under Pb stress conditions. Furthermore, the percentage of membrane stability index (MSI) showed a significant increase in esculin-treated flax plants compared to the stressed control (Table 1).

**Table 1:** Impact of different concentrations of Esculin “E50, E100 Mg L^−1^” on hydrogen peroxide (H_2_O_2_), singlet oxygen (O_2_^−•^), malonaldehyde (MDA), membrane stability index (MSI), electrolyte leakage (EL) and Lipoxygenase (LOX) activity of flax plants exposed to lead toxicity stress (L200 Mg/L). Each value is the mean of three replicates ± SE

Treatments	H_2_O_2_ (*n* mole/g FW)	O_2_^−•^ (*n* mole/g FW)	MDA (*n* mole/g FW)	MSI (%)	EL (%)	LOX Activity(1n mol of substrate oxidized/min)
L0	3.30 ± 0.026^d^	3.61 ± 0.04^d^	9.65 ± 0.24^d^	82.34 ± 0.173^a^	27.08 ± 0.35^d^	8.72 ± 0.02^d^
L 200 mg/L	6.29 ± 0.029^a^	7.40 ± 0.03^a^	15.39 ± 0.017^a^	51.7 ± 0.064^d^	38.5 ± 0.058^a^	18.38 ± 0.14^a^
L 200 mg/L + E 50 mg/L	5.12 ± 0.017^b^	6.66 ± 0.12^b^	13.11 ± 0.309^b^	57.4 ± 0.257^c^	34.6 ± 0.057^b^	16.20 ± 0.26^b^
L 200 mg/L + E 100 mg/L	4.95 ± 0.061^c^	5.79 ± 0.025^c^	11.60 ± 0.012^c^	63.0 ± 0.289^b^	30.9 ± 0.14^c^	14.26 ± 0.33^c^

Values with different letters in the column are significantly different at *p *< 0.05

### Mineral levels

The current data showed significant reductions in the levels of magnesium (Mg), potassium (K), calcium (Ca), zinc (Zn), and phosphorus (P) in flax shoots exposed to lead toxicity (Table 2). In contrast, sodium (Na) and lead (Pb) levels were significantly increased in the lead-stressed plants. Applying esculin at both concentrations (50 mg/L and 100 mg/L) markedly increased the levels of Mg, K, Ca, Zn, and P in Pb-stressed plants. Conversely, esculin treatment, especially at the higher concentration (100 mg/L), significantly reduced Na and Pb levels.

**Table 2:** Impact of different concentrations of Esculin “E50, E100 Mg L^−1^” on potassium, sodium, magnesium, calcium, phosphorous and lead contents as well as the sodium/potassium ratio of flax plants exposed to lead toxicity stress (L200 Mg/L). Each value is the mean of three replicates ± SE

Treatments	K (mg g^−1^)	Na (mg g^−1^)	Na/K	Mg (mg g^−1^)	Ca (mg g^−1^)	*P* (mg g^−1^)	Pb (mg g^−1^)
L0	26.73 ± 0.124^a^	6.11 ± 0.075^b^	4.37	6.23 ± 0.04^a^	9.60 ± 0.14^a^	13.27 ± 0.006a	0.024 ± 0.001d
L 200 mg/L	16.34 ± 0.29^d^	6.56 ± 0.04^a^	2.49	4.77 ± 0.05^d^	3.73 ± 0.06^d^	5.28 ± 0.04d	0.137 ± 0.001a
L 200 mg/L + E 50 mg/L	19.29 ± 0.19^c^	6.02 ± 0.08^b^	3.2	5.01 ± 0.03^c^	4.86 ± 0.10^c^	7.75 ± 0.23c	0.119 ± 0.003b
L 200 mg/L + E 100 mg/L	21.71 ± 0.07^b^	5.89 ± 0.03^b^	3.69	5.27 ± 0.04^b^	6.81 ± 0.07^b^	9.21 ± 0.03b	0.110 ± 0.0008c

Values with different letters in the column are significantly different at *p *< 0.05

### Plant growth and tolerance index (TI)

**Fig. 1:**
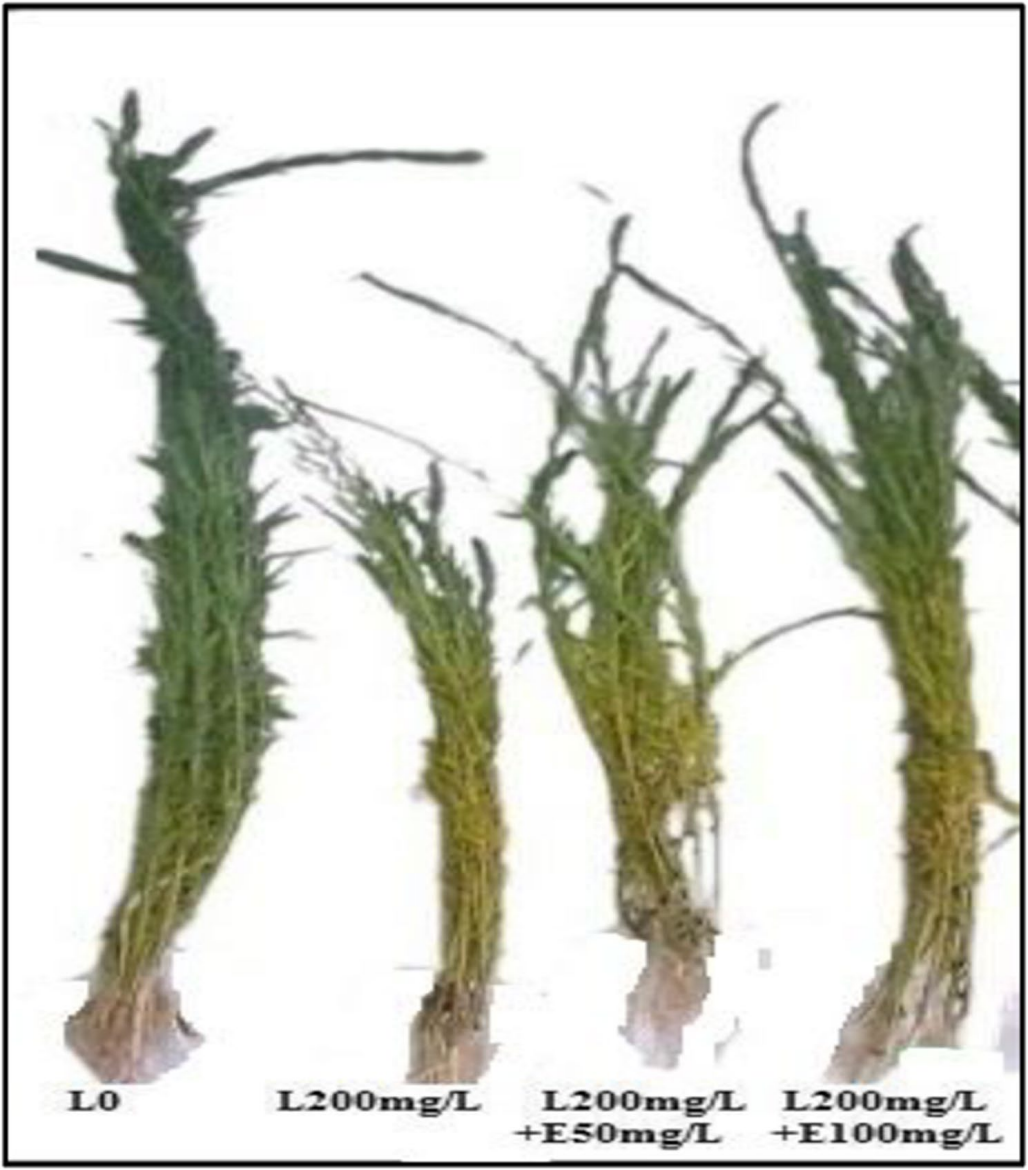
Impact of different concentrations of esculin “E50, E100 mg L^−1^” on growth of flax plants exposed to lead toxicity stress (L200 mg/L)

The growth parameters in terms of shoot length, shoot fresh weight, dry weight root length and root fresh weight of flax plants were significantly reduced under lead toxicity compared with the corresponding unstressed control (Table 3; Fig. 1). The tolerance index notably decreased under lead stress. Esculin foliar application at the two applied concentrations significantly enhanced the measured growth parameters as well as the tolerance index (TI) in Pb-stressed plants as being compared with stressed control (Table 3). Esculin at high concentration recorded 32.1%, 75%, 33.3%, 26.8% and 29.2% increased for shoot length, shoot fresh weight, dry weight root length and root fresh weight, respectively.

**Table 3:** Impact of different concentrations of Esculin “E50, E100 Mg L^−1^” on some growth parameters and tolerance index of flax plants exposed to lead toxicity stress (L200 Mg/L). Each value is the mean of three replicates ± SE

Treatments	Shoot length (cm)	Shoot Fresh weight (g)	Dry weight (g)	Root length (cm)	Root Fresh weight (g)	Tolerance Index (TI)
L0	36.33 ± 0.88^a^	1.57 ± 0.07^a^	0.41 ± 0.003^a^	10.33 ± 0.33^b^	0.37 ± 0.006a	100
L 200 mg/L	22.66 ± 0.33^d^	0.366 ± 0.03^c^	0.309 ± 0.002^c^	9.66 ± 0.33^b^	0.235 ± 0.008d	62.37
L 200 mg/L + E 50 mg/L	26.33 ± 0.33^c^	0.489 ± 0.01^c^	0.365 ± 0.007^b^	10.33 ± 0.66^b^	0.280 ± 0.002c	72.47
L 200 mg/L + E 100 mg/L	30.00 ± 0.57^b^	0.669 ± 0.01^b^	0.374 ± 0.01^b^	12.33 ± 0.33^a^	0.308 ± 0.002b	82.58

Values with different letters in the column are significantly different at *p *< 0.05

### Yield components

Esculin treatment significantly improved growth and yield parameters in flax plants under Pb stress during the mature stage (Table 4). Treated plants exhibited increased shoot length, technical stem length, and shoot dry weight compared to stressed control plants. Yield-related traits, including fruiting zone length, number of fruiting branches and fruits per plant, seed yield per plant, 1000-seed weight, and pod weight, were also markedly enhanced. Notably, these improvements were more pronounced at higher esculin concentrations, highlighting its dose-dependent efficacy in mitigating Pb stress effects.

**Table 4:** Impact of different concentrations of Esculin “E50, E100 Mg L^−1^” on yield components of flax plants exposed to lead toxicity stress (L200 Mg/L). Each value is the mean of three replicates ± SE

Treatments	shoot length (cm)	technical stem length (cm)	shoot dry weight (g)	fruiting zone length (cm)	number of fruiting branches/plant	the number of fruits/plant	seed yield/plant (g)	1000 seeds weight (g)	Pods weight (g)
L0	55.33 ± 0.57^ab^	44.33 ± 0.66a	3.04 ± 0.04a	11.00 ± 0.57^a^	6.33 ± 0.33ab	11.00 ± 0.57a	0.46 ± 0.002a	5.25 ± 0.04a	0.70 ± 0.02a
L 200 mg/L	45.66 ± 2.02^c^	39.00 ± 2.64b	2.08 ± 0.03b	6.67 ± 0.66^b^	4.66 ± 0.33b	8.33 ± 0.33b	0.26 ± 0.004c	2.74 ± 0.05d	0.30 ± 0.006c
L 200 mg/L + E 50 mg/L	51.33 ± 1.2^b^	42.00 ± 1.15ab	2.21 ± 0.02ab	9.33 ± 0.33^a^	6.00 ± 0.57ab	9.33 ± 0.33ab	0.28 ± 0.003c	3.13 ± 0.03c	0.32 ± 0.004c
L 200 mg/L + E 100 mg/L	56.33 ± 0.88^a^	45.33 ± 0.88a	2.79 ± 0.05a	11.00 ± 0.57^a^	7.33 ± 0.66a	9.66 ± 0.66ab	0.33 ± 0.011b	3.32 ± 0.03b	0.41 ± 0.002b

Values with different letters in the column are significantly different at *p* < 0.05

### Photosynthetic pigments

The photosynthetic pigment composition of flax leaves is differentially affected by lead (Pb) stress (Table 5). Chl *a*, Chl *b*, and total pigment content were significantly reduced in Pb-stressed leaves compared to the control, whereas carotenoid levels showed a significant increase. Treatment with esculin notably enhanced the levels of chlorophyll a, chlorophyll b, and carotenoids under Pb stress, with these increases being concentration-dependent (Table 5).

**Table 5:** Impact of different concentrations of Esculin “E50, E100 Mg L^−1^” on photosynthetic pigments, chlorophyll a (Chl *a*), chlorophyll b (Chl *b*) and carotenoids (car) µg/g fresh wt. of flax plants exposed to lead toxicity stress (L200 Mg/L). Each value is the mean of three replicates ± SE

Treatments	Chl a	Chl b	chla/chl b	Car	Total pigments
L0	1161.95 ± 1.8^a^	765.90 ± 1.7^a^	1.52 ± 0.001^b^	269.74 ± 3.4^c^	2197.58 ± 7.1^a^
L 200 mg/L	820.9 ± 0.36^d^	519.9 ± 2.67^d^	1.57 ± 0.007^a^	270.1 ± 0.85^c^	1611.1 ± 2.17^d^
L 200 mg/L + E 50 mg/L	838.3 ± 1.70^c^	560.3 ± 3.49^c^	1.49 ± 0.012^bc^	280.9 ± 0.21^b^	1679.5 ± 2.00^c^
L 200 mg/L + E 100 mg/L	860.6 ± 1.69^b^	580.5 ± 0.49^b^	1.48 ± 0.004^c^	302.3 ± 4.95^a^	1743.3 ± 3.75^b^

Values with different letters in the column are significantly different at *p* < 0.05

### Total soluble carbohydrates, free amino acids and IAA

**Fig. 2:**
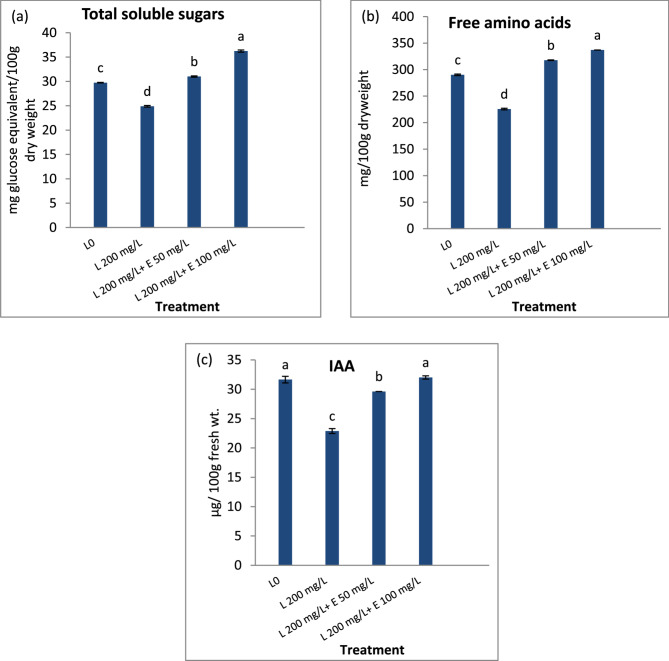
Impact of different concentrations of esculin “E50, E100 mg L^−1^” on content of (**a**) total soluble sugars (TSS), (**B**) free amino acids and (**c**) indole acetic acid (IAA) of flax plants exposed to lead toxicity stress (L200 mg/L). Each value is the mean of three replicates ± SE. Columns with different letters are significantly different at *p* < 0.05

The data revealed a significant reduction in total soluble carbohydrates, free amino acids, and indole acetic acid (IAA) content in Pb-stressed flax plants compared to unstressed controls (Fig. 2). Foliar application of esculin significantly improved these parameters in Pb-stressed flax plants relative to the stressed control. Such increments were significant in stressed leaves treated with 100 mg/L esculin.

### Antioxidants estimation of antioxidant enzyme activities, total phenols and proline content

The current results indicated that the exposure to lead (Pb) stimulated the antioxidant defense system activity in flax plants, as evidenced by a significant increase in the activities of superoxide dismutase (SOD), catalase (CAT), phenol peroxidase (POX), tyrosine ammonia lyase (TAL), and phenylalanine ammonia lyase (PAL) (Table 6). Furthermore, foliar application of esculin enhanced the antioxidant activities of all the aforementioned enzymes in Pb-stressed flax plants. This enhancement, along with the increased accumulation in antioxidant compounds such phenols observed, was found to be concentration-dependent. Additionally, the application of esculin significantly elevated the levels of total phenolic compounds and proline content in the Pb-stressed plants (Table 6).

**Table 6:** Impact of different concentrations of Esculin “E50, E100 Mg L^−1^” on the activities of superoxide dismutase (SOD), catalase (CAT), peroxidase(POX), tyrosine ammonia lyase (TAL), phenyl ammonia lyase (PAL) total phenolic compounds and proline contents of flax plants exposed to lead toxicity stress (L200 Mg/L). Each value is the mean of three replicates ± SE

Treatments	SOD (mM of H_2_O_2_ g^−1^ FW min.^−1^)	CAT (mM of H_2_O_2_ g^−1^ FW min.^−1^)	POX (amount of quinon g^−1^ FW min^−1^)	TAL (µg *p*-coumaric acid g^−1^min^−1^)	PAL (µg trans-cinnamic acid g^−1^min^−1^)	total phenolic compounds (mg g^−100^Dwt)	Proline (µg g^−1^ fresh weight)
L0	41.4 ± 0.12^d^	36.6 ± 0.2 ^d^	13.68 ± 0.0^d^	4.51 ± 0.08^d^	1.54 ± 0.012d	52.48 ± 0.65^d^	25.99 ± 0.2d
L 200 mg/L	44.24 ± 0.34^c^	51.05 ± 0.32^c^	22.59 ± 0.14^c^	8.39 ± 0.02^c^	3.4 ± 0.14c	54.56 ± 0.51^c^	40.63 ± 0.56^c^
L 200 mg/L + E 50 mg/L	49.76 ± 0.04^b^	57.39 ± 0.25^b^	31.17 ± 0.19^b^	9.4 ± 0.03^b^	4.1 ± 0.09b	61.24 ± 0.23^b^	48.25 ± 0.23^b^
L 200 mg/L + E 100 mg/L	59.21 ± 0.26^a^	66.43 ± 0.04^a^	35.90 ± 0.43^a^	9.91 ± 0.15^a^	4.56 ± 0.03a	77.03 ± 0.22^a^	50.74 ± 0.63^a^

Values with different letters in the column are significantly different at *p* < 0.005

### Seeds analysis

**Fig. 3:**
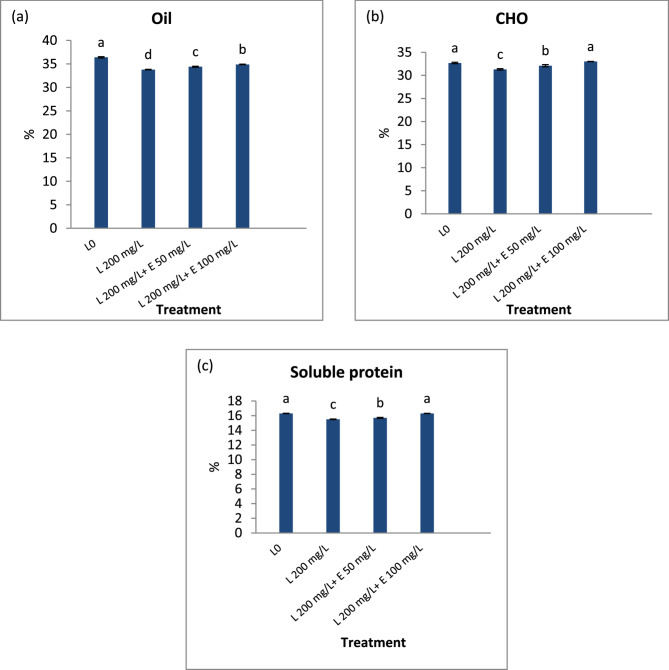
Impact of different concentrations of esculin “E50, E100 mg L^−1^” on (**a**) oil, (**b**) carbohydrate (CHO), and (**c**) protein contents of flax seeds exposed to lead toxicity stress (L200 mg/L). Each value is the mean of three replicates ± SE. Columns with different letters are significantly different at *p* < 0.05

The imposition of lead (Pb) resulted in a significant decrease in seed quality, as indicated by reduced oil (%), carbohydrate (%), and protein content (Fig. 3). However, foliar application of esculin at concentrations of 50 mg/L and 100 mg/L significantly improved these quality parameters in flax seeds.

### Straw yield

**Fig. 4:**
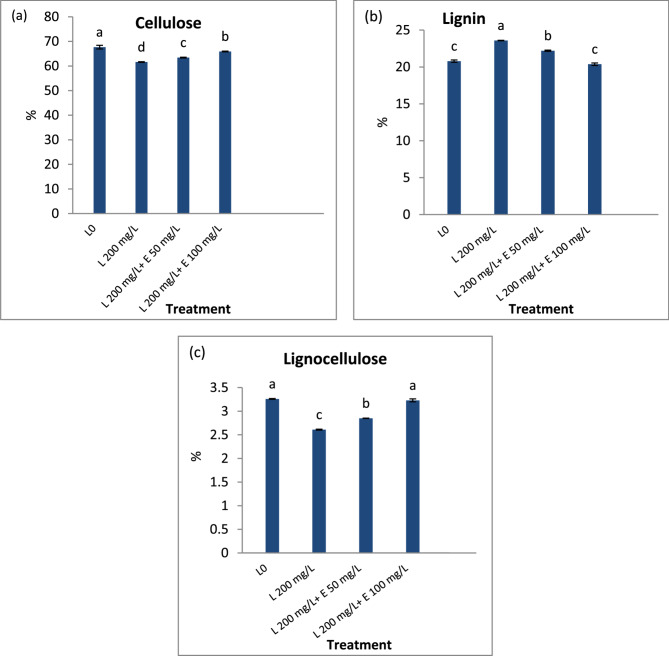
Impact of different concentrations of esculin “E50, E100 mg L^−1^” on (**a**) cellulose, (**B**) lignin and (**C**) lignocellulose content of flax fiber exposed to lead toxicity stress (L200 mg/L). Each value is the mean of three replicates ± SE. Each value is the main of three different replicates ± SE. Columns with different letters are significantly different at *p* < 0.05

The straw yield and fiber parameters in terms of fiber length, width, and fiber percentage were significantly improved by the foliar application of esculin (Table 7). The greatest percentage of increase was 6.6%, 35.5%, 29.4% and 4.9% for fiber length, fiber weight, straw yield/plant fiber % respectively, were recorded in plants subjected to high concentration of esculin exposed to Pb stress conditions. Lead significantly decreased and increased cellulose and lignin contents of flax fiber respectively (Fig. 4). Conversely, esculin application significantly increased and decrease cellulose and lignin contents in Pb-stressed flax as compared with stressed control.

**Table 7:** Impact of different concentrations of Esculin “E50, E100 Mg L^−1^” on fiber length, fiber weight, straw yield, and fiber percentage contents of flax plants exposed to lead toxicity stress (L200 Mg/L). Each value is the mean of three replicates ± SE

Treatments	Fiber length (cm)	Fiber wt/plant (g)	Straw yield/plant (g)	Fiber %
L0	53.6 ± 0.3a	0.25 ± 0.008^d^	1.47 ± 0.04^c^	17.22 ± 1.1^a^
L 200 mg/L	45.6 ± 1.2c	0.31 ± 0.01^c^	1.72 ± 0.03^b^	18.22 ± 0.68^a^
L 200 mg/L + E 50 mg/L	47.3 ± 0.8bc	0.35 ± 0.003^b^	1.84 ± 0.08^b^	19.27 ± 0.78^a^
L 200 mg/L + E 100 mg/L	48.6 ± 0.3b	0.42 ± 0.003^a^	2.2 ± 0.02^a^	19.08 ± 0.05^a^

Values with different letters in the column are significantly different at *p* < 0.05

## Discussion

Lead (Pb) contamination poses a significant threat to crop quality, yield, and human health through its entry into the food chain. The mechanisms of flax tolerance to Pb toxicity remain largely unexplored [61]. In this study, the exposure of flax to Pb stress induced substantial reactive oxygen species (ROS) accumulation, including H_₂_O_₂_ and O_₂_^−•^, compared to unstressed plants (Table 1). This ROS flow triggered oxidative stress, evidenced by elevated lipid peroxidation products (MDA), loss of membrane integrity, and disrupted homeostasis, as indicated by increased electrolyte leakage (EL) and decreased membrane stability index (MSI) (Table 1). The hyperaccumulation of ROS disturbs the homeostasis of cells, causing lipid peroxidation and membrane damage, consequently increasing membrane leakage and may cause cell death [17, 62]. High Pb concentrations causes phytotoxicity of cell membrane and unusual mechanisms such as alteration in cell membrane permeability, interaction between sulphydryl (-SH) groups and cations as well as the reaction with phosphate groups and active groups of ADP and ATP [63]. Enhanced lipoxygenase (LOX) activity under Pb stress further exacerbated lipid peroxidation and MDA accumulation (Table 1) [64].

The results of the present investigation showed that Pb stress significantly reduced the uptake of essential nutrients, including K⁺, P, Mg, and Ca, while increasing Na⁺ content and Na⁺/K⁺ ratios (Table 2). These alterations disrupted membrane homeostasis, adversely impacting plant metabolism, growth, and productivity (Table 3; Fig. 1) and (Table 4). Probably, Pb toxicity impairs the uptake of critical elements such as Mg, K⁺, Ca, P, Mn, Zn, Cu, and Fe³⁺, potentially due to ionic size similarities or modifications in membrane enzyme activity and structure. Notably, the efflux of K⁺ from roots, attributed to the sensitivity of K⁺-ATPase and -SH groups in membrane proteins to Pb, further underscores the toxic effects of Pb stress [65].

In addition, Pb contamination resulted in a significant decrease in photosynthetic pigments chlorophyll a and b (Tale 5) which may be attributed to an increase in its degradation or a decrease in its synthesis. The photosynthetic pigments may be subjected to degradation by the effect of accumulated ROS also, Pb may accelerate the degradation of chlorophyll by increased activity of chlorophyllase enzyme [66]. On the other hand, Pb could inhibit the biosynthesis of chlorophyll by inhibiting α-amino laevulinate dehydrogenase which is an essential enzyme in biosynthesis of chlorophyll [67]. The decrease in chlorophyll content in Pb-stressed flax was associated with a significant decrease in Mg ions concentration (Table 2) as compared with unstressed control. Mg is an important microelement that has a structural role in chlorophyll as it occupies a central position in the porphyrin head of the chlorophyll molecule [68, 69]. Moreover, Pb could replace magnesium in chlorophyll molecules, resulting in less chlorophyll concentration [70]. The reduction in photosynthetic pigments under Pb stress is expected to reduce the rate of photosynthesis, as evidenced by the decline in primary photosynthetic products, including total soluble sugars and free amino acids, in Pb-stressed flax compared to the unstressed control (Fig. 2a & b). In addition, the decrease in Mg of lead-stressed flax (Table 2) also participate in the reduction of photosynthetic rate as it is required for activation of various enzymes involved in carbohydrate metabolism such as ribulose-1,5-bisphosphate carboxylase/oxygenase as well as kinases enzymes [71]. Also, Pb contamination resulted in a significant decrease in IAA (Fig. 2c), which is an essential growth promoter hormone that displayed a main role in cell division and elongation [72], so its reduction impaired flax growth and yield parameters under lead contamination (Tables 3 and 4). Although, Pb is non-essential element for the plant growth and metabolism, once enters the plant it stimulates a wide range of adverse impacts on several metabolic processes such as photosynthesis, enzyme activity inhibition, altered mineral nutrition, hormonal changes, and changes in membrane permeability, and absorption of water [65, 73–75]. The current results are in line with that of Bharwana et al., [76] who proved that lead treatment significantly reduced photosynthetic pigments content, growth parameters and biomass of cotton plant. Many studies have been proven that lead contamination adversely affect plant growth and biomass [77–80].

Exposure to Pb and other heavy metals typically induces oxidative stress in plants. The activation of the antioxidant defense system is a crucial adaptive mechanism to mitigate oxidative damage under heavy metal stress [81–83]. In the present study, Pb-stressed flax plants exhibited significantly increased activity levels of antioxidant enzymes, including SOD, CAT, POX, LOX, PAL, and TAL (Table 6), as well as elevated levels of antioxidant compounds such as carotenoids (Table 5), phenolic compounds, and proline, compared to unstressed plants (Table 6). The increase in phenolic compounds was related to enhanced PAL and TAL enzyme activity. PAL plays a pivotal role in synthesizing p-coumaroyl CoA, a key precursor in the biosynthesis of diverse phenolic compounds [84]. Several studies have also documented the upregulation of antioxidant enzymes in response to Pb stress across various plant species, including *Typha latifolia*, *Phragmites australis*, *Lemna minor L.*, and *Juncus effusus L.* [85–87].

Esculin exhibits potent antioxidant activity, primarily through its capacity to scavenge reactive oxygen species (ROS) [21, 22, 88]. Consistent with our findings, esculin effectively mitigated ROS accumulation, as evidenced by the significant reductions in hydrogen peroxide (H₂O₂) and superoxide anion (O₂^−•^) levels in esculin-treated, Pb-stressed plants compared to untreated Pb-stressed controls (Table 1).

In addition, esculin stimulated the endogenous antioxidant defense system of flax under Pb stress condition as it evident by the significant increase in some antioxidant enzymes such as SOD, CAT, POX, PAL and TAL enzyme (Table 6). These antioxidant enzymes participated in the scavenging of ROS, SOD catalyze the conversion of O_2_^−•^ into O_2_ and H_2_O_2_ [89], then H_2_O_2_ could be eliminated by peroxidase [90] and catalase enzyme that utilizes H_2_O_2_ into water and molecular oxygen [91]. The stimulation of antioxidant enzymes by esculin may result from increased gene expression, as reported by Ju et al., [25], who stated that esculin upregulates the expression of antioxidant proteins such as glutathione reductase, superoxide dismutase, and glutathione peroxidase. This led to reduced lipid peroxidation caused by ROS, evidenced by a significant decrease in MDA content in esculin-treated flax compared to untreated Pb-stressed plants (Table 1). MDA, a reliable biomarker for oxidative stress alleviation [92], reflects reduced oxidative damage, improved membrane integrity, lower electrolyte leakage (EL), and increased membrane stability index (MSI) (Table 1). Previous studies have shown that esculin enhances antioxidant defenses by reducing ROS and MDA levels [24] and boosts activities of CAT, SOD, and POX under salt stress [93]. Consistently, our findings indicate that the decrease in MDA in esculin-treated flax is accompanied by reduced activity of the LOX enzyme compared to untreated plants (Table 1).

Esculin, a glycosidic coumarin containing a carbohydrate moiety, likely contributes to maintaining membrane integrity and enhancing nutrient uptake. This is demonstrated by the significant increases in K, P, Ca, and Mg content, along with a marked reduction in Na content, in Pb-stressed flax treated with esculin compared to untreated stressed plants (Table 2). These changes support the restoration of the electrochemical gradient across the plasma membrane, essential for membrane homeostasis, secondary transport of organic and inorganic molecules, and osmotic regulation [93]. Consequently, these effects significantly improved the growth and yield parameters of flax under Pb stress (Tables 3 and 4).

Reducing Pb uptake is a key strategy to enhance plant tolerance to Pb toxicity [94]. The current study demonstrates that esculin significantly decreased Pb content in Pb-stressed flax compared to stressed controls (Table 3). This effect may be attributed to the azomethine and phenolic hydroxyl groups present in the coumarin moiety of esculin [95], as coumarin-derived ligands are known to form complexes with various d-block metals [96]. Additionally, the carbohydrate moiety and hydroxyl groups in esculin likely enhance its chelating and adsorbent properties, reducing Pb uptake by plants. Coumarin derivatives, classified as oxygen-type ligands, have also been utilized in wastewater treatment to remove cationic trace metals such as Pb [97].

Esculin alleviated Pb toxicity by stimulating the synthesis of endogenous phytohormones, notably increasing IAA levels in Pb-stressed flax compared to untreated plants (Table 4). Auxins, such as IAA, are known to modulate membrane properties, mitigating heavy metal toxicity. Combined application of Pb and IAA has been shown to reduce membrane disruption and alleviate heavy metal stress [98]. The increase in IAA concentration in esculin-treated plants may result from enhanced IAA biosynthesis, potentially stimulated by the glycoside moiety of esculin, and/or reduced IAA degradation due to esculin’s antioxidant properties [93]. As a growth promoter, IAA stimulates cell division and elongation [72], contributing to improved growth and yield parameters in esculin-treated flax under Pb stress compared to untreated controls (Tables 3 and 4).

Esculin mitigates Pb-induced oxidative stress by enhancing the accumulation of non-enzymatic antioxidants, including carotenoids (Table 5), phenolic compounds, and proline (Table 6). The increased phenolic content in esculin-treated flax under Pb stress may result from enhanced activity of PAL and TAL enzymes compared to stressed controls (Table 6). Carotenoids contribute to light harvesting, expand the absorption spectrum, protect photosynthetic pigments by dissipating excess energy as heat, and scavenge harmful oxygen species like singlet oxygen (¹O_₂_) [99–102]. This carotenoid increase coincides with a significant rise in chlorophyll a and b levels (Table 5), accelerating photosynthesis, as evidenced by higher total soluble sugar and free amino acid concentrations, key photosynthetic products (Fig. 2a & b).

Osmoregulation, a critical detoxification strategy for Pb toxicity, relies on osmolyte accumulation, including sugars, amino acids, and proline [103], which scavenge ROS and mitigate Pb-induced damage [104]. Additionally, amino acids can chelate Pb and sequester it in the tonoplast, effectively detoxifying Pb through chelation, a key mechanism for heavy metal stress alleviation [105].

The acceleration of photosynthesis enhances the production of both primary and secondary metabolites in plants [106]. Esculin significantly increased total phenolic compound levels (Table 6) compared to untreated plants. Phenolic compounds exhibit strong antioxidant properties due to their ability to chelate metals through carboxyl and hydroxyl groups, particularly binding copper and iron [107]. This chelation inhibits the Fenton reaction, a major source of reactive oxygen species (ROS) [108, 109].

The enhancement of photosynthesis, along with increased primary metabolites, soluble sugars, and free amino acids (Fig. 2a & b), contributes to improved seed quality, as evidenced by significant increases in seed weight, and percentages of carbohydrates, proteins, and oil (Fig. 3) [110]. Improvements in chlorophyll content, photosynthesis, IAA levels, and nutrient uptake metabolic changes induced by esculin markedly enhanced the growth and straw yield of esculin-treated flax compared to Pb-stressed untreated flax. Esculin also improved straw quality, significantly increasing fiber length, width, and percentage (Table 7), while enhancing cellulose content and reducing lignin levels compared to untreated plants (Fig. 4). Conversely, Pb stress reduced chlorophyll content, photosynthesis, and nutrient uptake, leading to stunted growth and inferior straw quality (Table 7) and (Fig. 4). Esculin treatment increased flax tolerance to Pb, as indicated by a higher Tolerance Index (TI), particularly at elevated Pb concentrations, compared to stressed controls (Table 3). TI, a measure of plant growth under metal stress relative to controls, is widely used to assess stress resilience [72]. Reduced TI has been observed in tobacco roots exposed to 4–5 mM Pb²⁺ [111] and in water spinach with increasing Pb concentrations [112].

## Conclusion

This study confirms that esculin, a plant-derived coumarin glycoside, significantly mitigates lead (Pb) toxicity in *Linum usitatissimum* (flax) by enhancing growth performance, yield components, and antioxidant defense mechanisms. Esculin can scavenge ROS and upregulate antioxidant enzymes such as superoxide dismutase (SOD), catalase (CAT), and glutathione reductase (GR), highlighting its potential as a bio-protective agent. Esculin presents a sustainable and eco-friendly strategy for mitigating heavy metal stress in crops. Its practical applicability in phytoremediation and enhancing agricultural resilience offers significant potential for improving productivity in contaminated soils and promoting sustainable crop management.

Further research on esculin should focus on elucidating its precise molecular mechanisms in stress mitigation, particularly under heavy metal toxicity. Investigating its interactions with key signaling pathways, gene expression profiles, and antioxidant systems in various crops could enhance its application in sustainable agriculture. Field trials and formulation development are also needed to evaluate its effectiveness under real-world conditions. Additionally, exploring its synergistic effects with other bioactive compounds may expand its role in phytoremediation and crop resilience strategies.

## Data Availability

Data is provided within the manuscript.
